# A Report from a Community-Centric Cancer Control Approach in the Post-Conflict Northern Province of Sri Lanka

**DOI:** 10.3390/ijerph22101492

**Published:** 2025-09-27

**Authors:** Abiola N. Dosumu, Antony J. Thanenthiran, Ganeshamoorthy Sritharan, Thanuja Mahendran, Rajendra Surenthirakumaran, Kandasamy Sithamparanathan, Stephanie Asence, Kathleen M. Decker, Sri Navaratnam

**Affiliations:** 1 Health Service Research Lab, Paul Albrechtsen Research Institute CancerCare Manitoba, Winnipeg, MB R3E 0V9, Canadakdecker@cancercare.mb.ca (K.M.D.); 2 Department of Surgical Oncology, Jaffna Teaching Hospital, Jaffna 40000, Sri Lanka; jthana83@gmail.com (A.J.T.); srieye@yahoo.com (G.S.);; 3 Department of Community and Family Medicine, Faculty of Medicine, University of Jaffna, Jaffna 40000, Sri Lanka; surenthirakumaran@gmail.com; 4 Department of Drama and Theatre, University of Jaffna, Jaffna 40000, Sri Lanka; 5 Department of Community Health Sciences, Rady Faculty of Health Sciences, Max Rady College of Medicine, University of Manitoba, Winnipeg, MB R3T 2N2, Canada; 6 Department of Internal Medicine, Rady Faculty of Health Sciences, Max Rady College of Medicine, University of Manitoba, Winnipeg, MB R3T 2N2, Canada

**Keywords:** delivery of healthcare, community health services, health personnel, public health infrastructure, cancer risk factors, early detection of cancer, cancer control, resource-limited setting, Sri Lanka

## Abstract

Late-stage cancer diagnoses of prevalent cancers are increasing in the Northern Province of Sri Lanka, a region currently rebuilding its healthcare system after a prolonged civil war. In this region, cancer prevention services are limited. We describe a community-centric approach to cancer education and prevention as a strategy to cancer control in this rural, post-conflict region. Nursing students were trained as Community Cancer Educators (CCEs), equipping them with essential knowledge about cancer symptoms, risk factors, and the importance of early detection. The training also included creative methods such as dance and drama to help CCEs communicate cancer-related messages in an engaging and culturally relevant manner. These CCEs supported the oncologist-led community health camps in delivering cancer education and screening directly to community members within their community. We planned the health camps in collaboration with the existing community-based public health system for better outreach. Feedback from community participants and healthcare providers suggests that this community-centric approach can improve cancer awareness, encourage participation in population screening, and support early cancer detection. This approach could strengthen community engagement and contribute to more equitable access to prevention and screening services in rural, post-conflict settings with limited healthcare infrastructure.

## 1. Introduction

Cancer incidence in Sri Lanka has doubled in the last 25 years, making it the second leading cause of hospital mortality nationally [1,2]. In Sri Lanka, the National Cancer Control Program (NCCP) was established in 1980 to coordinate cancer prevention and control efforts across the country [2]. Over the years, several health promotion interventions have been implemented to improve awareness and early detection [3]. These interventions have focused on commonly diagnosed cancers including oral, breast, and cervical cancers. Interventions include the Well Woman Clinic (WWC) program, social marketing campaigns, and school-based programs [3,4,5]. Despite these efforts, Sri Lanka currently reports about 103 new cancer cases daily, totaling about 37,753 cases per year annually [6]. The NCCP reports that over 40% of breast and cervical cancers, as well as 70% of oral cancers, are diagnosed at advanced stages (stages 3 and 4) [6,7]. This complicates treatment, leads to poor health outcomes, and decreases survival rates.

CancerCare Manitoba (CCMB), whose overarching vision is “leading the way in cancer control” has collaborated with the University of Jaffna for nearly a decade to advance cancer prevention and control strategies in Sri Lanka’s Northern Province. The collaboration began with efforts to establish a cancer registry in the region by conducting a retrospective, cross-sectional study of hospital cancer cases from 2015 to 2018. The study identified 3266 cases, with the highest prevalence among individuals aged 60 to 69 years (32.7%). Oral cancer was the most common cancer in the Northern Province. This differs from the national data, which reports breast cancer as the most prevalent cancer [7], emphasizing the need for region-specific cancer prevention and control strategies.

In many low- and middle-income countries (LMICs) including Sri Lanka, screening programs are available for oral, breast, and cervical cancer [8]. However, these programs are often concentrated in specialized urban centers and are difficult for rural populations to access due to barriers such as lack of awareness, financial constraints, and travel requirements [9,10,11]. Research shows that rural populations often have lower cancer screening rates and are diagnosed at a more advanced stage, contributing to poorer outcomes [12,13,14]. This is evident in Sri Lanka’s Northern Province, home to approximately 1.2 million people, of whom 83.4% live in rural areas [15]. This region was severely affected by 16 years of prolonged civil war and is currently rebuilding its healthcare infrastructure [16,17,18]. As a rural, post-conflict setting, the Northern Province faces multiple barriers to healthcare, including weak health infrastructure, limited specialist services, long travel distances, poverty, and low health literacy [19]. Addressing these challenges requires both strengthening healthcare infrastructure and community engagement to improve acceptance and reach in underserved populations [20].

Community-focused approaches have been used in similar LMICs to improve early cancer detection and increase cancer awareness [20,21,22]. For example, in rural India, a population-based cancer screening program that trained community volunteers and health activists led to early diagnosis in nearly half of oral cancer cases [23]. Similarly, community health workers across South Asia have been instrumental in promoting breast and cervical cancer screening and delivering community cancer education in underserved populations [24,25].

Building on these examples, we designed a community-centric cancer education and screening project in the Northern Province of Sri Lanka. The long-term goals are to increase awareness, promote early detection, and improve equitable access to cancer prevention services. This report describes the early phase of the project, highlighting local partnerships and using culturally tailored strategies to engage the rural, post-conflict populations with limited access to healthcare services.

## 2. Materials and Methods

### 2.1. Project Overview

**Figure 1:**
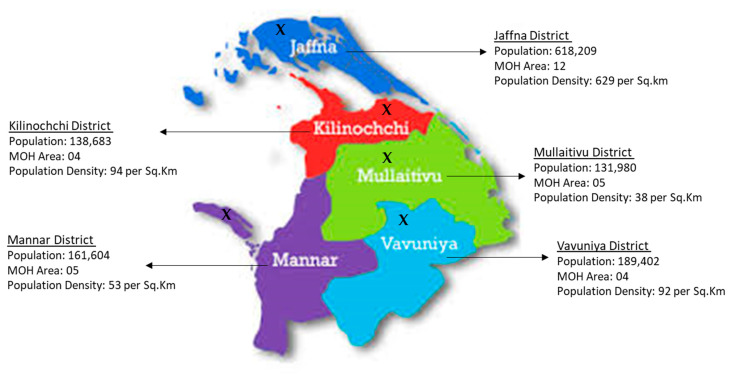
District profile of Northern Province of Sri Lanka, with X showing the location of the health camp in each province [26].

This community-centric cancer education and screening project was conducted across all five districts of Sri Lanka’s Northern Province: Jaffna, Kilinochchi, Mullaitivu, Mannar, and Vavuniya (Figure 1). This initial phase tested a community-centric cancer control approach by delivering cancer education and basic screening services directly to community members in accessible venues such as community halls and schools. The project consisted of six one-day, oncologist-led community health camps hosted at accessible community venues. These health camps were conducted between June 2022 and July 2023. The camps provided culturally tailored education using dance and drama, followed by on-site screening for oral and breast cancers.

The project design was based on Sri Lanka’s existing public health system, which is divided into preventive and curative services. Preventive services are delivered through the Medical Officers of Health (MOHs), who oversee community-focused public health services in each district. MOHs are public health medical doctors and are supported by public health staff. MOHs and staff are responsible for coordinating outreach and ensuring services reach underserved rural areas [27]. The project team collaborated closely with MOHs and local community leaders to select culturally appropriate and logistically feasible venues and schedule for each health camp.

The project was carried out in three stages: planning and stakeholder engagement, training of Community Cancer Educators (CCEs), and community cancer education and screening delivery.

### 2.2. Stage 1: Planning and Stakeholder Engagement

The planning phase, which took place during the first six months of the project, was focused on securing institutional support and aligning community engagement strategies across all five districts of the Northern Province. It involved collaboration between CCMB and Sri Lanka partners, including oncologists from the Centre for Early Detection of Cancer (CEDC) Jaffna and Department of Community Medicine and Department of Drama and Theatre at the University of Jaffna. These discussions focused on improving community-level cancer awareness and early detection, while identifying practical ways to leverage existing health infrastructure to support the project. Meeting outcomes were formally communicated to the Northern Provincial Director of Health Services and the five Regional Directors of Health Services to ensure alignment with provincial health planning. The project team then partnered with Cancer Aid North East (CANE), a local non-governmental organization focused on cancer awareness to support and coordinate community efforts at the district level. CANE facilitated connection with MOHs, public health officers, and community leaders across districts. These district stakeholders assisted with identifying accessible venues and arranging logistics such as transportation to the venue. The project sponsor covered costs related to transportation and other essential logistics. Health camps were intentionally scheduled in neutral, familiar, non-clinical community spaces such as schools and community halls. These venues were selected to help participants feel comfortable and reduce the stigma around cancer and clinical settings. Community engagement was further strengthened by involving locally based nursing students such as CCEs.

### 2.3. Stage 2: Training of Community Cancer Educators (CCEs)

Twenty-five nursing students from the Nurse Training School, Jaffna, were selected to work as CCEs based on their interest in community health education and their willingness to support their communities. The training took place over one day, and content was developed in accordance with the NCCP of Sri Lanka to ensure alignment with national cancer prevention guidelines.

The morning session focused on cancer education, led by three oncologists from the CEDC and a psychiatrist from the Teaching hospital, University of Jaffna. This included cancer development, behavioral and lifestyle-related risk factors for cancer, prevention strategies relevant to the local context, solutions to supporting individuals experiencing emotional distress related to cancer screening, and communication strategies for sensitive conversations.

The afternoon session, facilitated by the University of Jaffna’s Drama and Theatre Action Group, focused on culturally tailored communication strategies. The session emphasized the use of Tamil dance and drama to share cancer information in familiar and engaging ways. The training prepared CCEs to support health professionals and effectively communicate cancer messages in a manner that was relevant and easily understood by the community members.

### 2.4. Stage 3a: Health Camp Delivery—Community Education

The health camps’ education session was open to all community members, providing an opportunity for individuals to learn about cancer prevention, and were oncologist-led. Each camp brought together a multidisciplinary team including 3 onco-surgeons (2 males, 1 female), 2 oral-maxillofacial surgeons, and between 9 and 15 female cancer nurses from Teaching Hospital Jaffna. Also in attendance were 15–20 CCEs, MOH staff, a community project coordinator, representatives from the University of Jaffna’s Drama and Theatre Action Group, and local volunteers who supported with logistics and venue setup.

Planning and outreach for the camps were coordinated with CANE and CCEs, who worked closely with village leaders to invite community members through community gatherings and word of mouth. Transportation was arranged for participants from remote areas to ensure equitable access to the health camp. Each health camp began with a cultural performance by the CCEs and the Drama and Theatre Action Group featuring the Tamil dance to create a welcoming environment, ease anxiety, and encourage community engagement.

The oncologist-led education session described lifestyle-based risk factors for cancer including tobacco use, betel quid chewing, and alcohol consumption. They emphasized how healthier choices can reduce cancer risk and how early detection improves treatment outcomes and survival. The screening process was described, and a question-and-answer session was conducted before the start of screening.

### 2.5. Stage 3b: Health Camp Delivery—Screening

Registered individuals 25 years of age and older, who provided verbal consent and completed a brief sociodemographic questionnaire, were eligible for screening. Consent was obtained to confirm their voluntary participation in the screening process, including a clinical examination and follow-up communication if required. A questionnaire that captured basic demographic details, lifestyle-related cancer risk factors, and family history of cancer was completed to understand those who participated and help plan future community interventions.

Screening participants were divided into smaller groups. For each group, a cancer nurse and CCEs discussed cancer prevention, explained the screening process for oral and breast cancer, and answered participants’ questions to ensure they felt informed and comfortable. Screening was then provided by the onco-surgeons, oral-maxillofacial surgeons, and cancer nurses.

- Oral-related cancer screening included a visual inspection with handheld lights and palpation of the head, neck, and cervical lymph nodes. Intraoral examinations were conducted with white light, and findings were recorded as normal or abnormal. Abnormalities checked during oral screening included visible lesions, non-healing ulcers lasting more than two weeks, palpable lumps, or swelling in the jaw or neck. This method has demonstrated 87% sensitivity and 50% specificity in detecting potentially malignant oral disorders [28]. It is also effective in reducing oral cancer mortality when used in population-based screening in LMICs [28,29,30].
- Breast cancer screening was conducted using a clinical breast examination (CBE) performed by trained female cancer nurses. CBE is an inexpensive screening method particularly suitable for LMICs [31]. Evidence shows that it can improve chances of successful treatment and survival by enabling earlier diagnosis [32]. Abnormalities checked included palpable lumps with irregular borders, asymmetry between breasts, skin dimpling, and nipple retraction.

Screening participants with abnormal findings were scheduled for follow-up diagnostic evaluation at the CEDC. Each referred participant was assigned to a CCE to provide guidance. The CCE explained the importance of follow-up, outlined the next steps, and ensured the participant understood the date, time, and location of the appointment.

### 2.6. Data Collection

Data collection was integrated into the screening process.

## 3. Results

A total of 847 individuals participated in screening in the health camps (Supplementary Table S1). Two hundered and thirty-seven indivduals (28%) participated in Vavuniya, 181 in Jaffna (21%), 168 in Kilinochchi (20%), 131 in Mannar (15%), and 130 in Mullaitivu (15%). Among the screening participants, five hundred ninety-four (70%) identified as female. Participants’ ages ranged from 24 to 98 years of age, with an average age of 49 years of age. The most reported occupation was housewife (*n* = 199, 23%), followed by craft and trade worker (*n* = 132, 16%), and agricultural worker (*n* = 67, 7%). Forty-four percent (*n* = 378) of participants did not report an occupation.

Thirty-seven percent of the screened participants (*n* = 313) reported chewing bethel quid, which is frequently chewed with slaked lime and/or tobacco and has been linked to oral cancer [33,34,35]. Other behavioral risks factors were reported less frequently, with 8.6% of participants (*n* = 73) reporting alcohol consumption and 5.4% (*n* = 46) reporting tobacco smoking. Occupational risk factors reported included radiation exposure (*n* = 49, 5.8%) and industrial chemical exposure, which was less than 1%. A family history of cancer was reported by 146 participants (17%) and included breast cancer (*n* = 22, 2.6%) and oral cancer (*n* = 18, 2.1%). Among these, 78 individuals reported that the affected relative was a first-degree family member such as a parent, sibling, or child.

Out of the 847 participants screened, 94 (11%) were referred for further diagnostic assessments at CEDC Jaffna. Abnormalities from oral screening had the highest number of referrals. Some referrals were also made based on reported symptoms such as difficulty swallowing, despite the absence of visible clinical signs. Data were not collected after the end of the health camp, as this initial phase focused on delivering education and screening within the community.

Participants reported feeling more informed about cancer risks and the importance of early detection (Supplementary Section S2), although awareness was not formally measured. They credited the dance and the CCEs for delivering cancer prevention information in a relatable and memorable manner. Participants valued the convenience of receiving services directly in their communities, as it reduced access barriers to healthcare services. Healthcare workers expressed support for this proactive approach and supported bringing the hospital to the community. Bringing the hospital to the community was how one of the cancer nurses described the strategy of delivering specialist-led care and education in an accessible, familiar, community setting.

## 4. Discussion

The long-term goal of this project is to improve community cancer awareness and prevention through education and the promotion of early detection in a post-conflict setting with limited healthcare infrastructure. We conducted oncologist-led community health camps that offered both education and screening, using culturally tailored approaches to actively engage participants. We demonstrated that delivering cancer prevention services directly in the community allowed us to reach underserved populations to identify early signs of cancer. Education was a central focus, as it is often the first and most critical step in effective cancer prevention efforts [36]. Evidence shows that culturally tailored educational programs are more effective in encouraging behavioral change and increasing involvement in cancer prevention efforts [37,38]. This has been observed more in low-resource settings and among people with limited health literacy [39,40,41].

The majority of the screening participants were female. This may be partially explained by the longstanding presence of Well Woman Clinics (WWCs) in Sri Lanka. WWCs were created to raise awareness about women’s preventive healthcare and provide screening for selected non-communicable diseases including cancer [42]. However, actual service utilization remains low. A district-level study [43] showed that 60% of women were aware of the services of the WWC, but only 18.5% used the service. The existing knowledge of such services may have made women more receptive to community-centric health camps provided in this project, despite the underutilization of fixed clinics, supporting the fact that education about cancer risk factors and screening is critical. One plausible reason for lower participation by men is that, as primary income earners, men may be unable to attend a full-day camp if it results in lost wages. Women, particularly housewives, had greater flexibility in their daily schedules, which may have contributed to the observed participation rate. In many South Asian settings, women are more likely to engage in community health activities, especially when led by local female educators or health staff [44]. This possibly reflects cultural norms that position women as primary caretakers, responsible for managing their family’s health [45]. Their involvement often extends beyond personal care, as they frequently share health information within their social networks and encourage others to seek care [46]. This presents a future opportunity to engage women as active disseminators of cancer knowledge in their community.

A key behavioral risk factor reported by over one-third of participants was betel quid chewing. Betel quid, commonly used in South Asia for its stimulant effects, typically consists of areca nut, slaked lime, and sometimes tobacco, wrapped in a betel leaf [47]. Betel quid chewing has been strongly associated with oral cancer [33,34,35]. In response to this risk, the Sri Lankan Ministry of Health has issued a national ban on the use of betel quid, tobacco, and areca nut in public institutions [48]. Yet the habit remains widespread. Public awareness campaigns have used leaflets, posters, stickers, and TV spots to inform the population about the dangers of betel quid chewing and its connection to oral cancer [49]. While these campaigns have increased awareness, whether they have led to meaningful or lasting changes in behavior remains unclear. This uncertainty points to the need for a more community-centric approach to cancer control that delivers culturally tailored education, engages community members, and incorporates systems for evaluating long-term impact. One strength of the approach used in this project was the ability to leverage existing public health systems, which reduced the need for additional healthcare resources. Through this process, we learned that collaborating with community organizations and community leaders helped to improve the cultural relevance of health outreach and build the trust needed in a post-conflict setting. Early engagement with local authorities, including community leaders, is as important as technical expertise when delivering cancer control activities. This approach may contribute to sustained behavior change in the future, which is an outcome that national media campaigns have struggled to achieve.

### 4.1. Limitation

While we report encouraging evidence of this community-centric approach to cancer control, we acknowledge several limitations and areas for further exploration. The project did not examine follow-up or treatment outcomes; hence, we are unable to assess the long-term impact of the intervention on health outcomes. Additionally, cancer risk factors and screening awareness were not formally measured. Therefore, we are unable to quantify changes in knowledge or behavior attributable to the intervention. Participation rates could not be calculated, as information about the number of potentially eligible individuals in each district was not collected.

### 4.2. Future Work

Future work includes the design and implementation of an intervention guided by the RE-AIM framework. Baseline data on cancer burden and district-level eligibility will inform the evaluation, with a particular focus on assessing the intervention’s reach and effectiveness. We will also include follow-up information such as the number of individuals who are diagnosed with cancer, treatment completion rates, and survival rates to assess the impact of the intervention. We are also working with MOHs to formalize the role of community health volunteers, including CCEs, to ensure the sustainability of the model of care at the community level. This approach has been successful in other LMICs [50], such as India, where trained community health workers achieved diagnostic accuracy in oral cancer screening comparable to that of dentists [51].

## 5. Conclusions

A community-centric approach to cancer education and screening was feasible in Sri Lanka’s underserved Northern Province. Culturally tailored health camps, designed with sensitivity to post-conflict realities, engaged hundreds of rural community members, most of whom had never participated in cancer screening. In addition to increasing access to cancer services, the project contributed to building a local capacity that can promote sustainable behavior change as part of long-term cancer prevention efforts. Decentralizing cancer education and screening by bringing prevention to the people, rather than requiring people to travel to central facilities, is a promising strategy for cancer control. If integrated into the national cancer control strategy with clear evaluation metrics, this community-centric approach can serve as an equitable and scalable model for cancer prevention and early cancer detection in underserved populations.

## Data Availability

The data is the property of the authors and can be available upon request.

## Supplementary Materials

The following supporting information can be downloaded at https://www.mdpi.com/article/10.3390/ijerph22101492/s1, Table S1: Characteristics of participants at community health camps in Sri Lanka’s Northern Province, by district.; S2: Testimonials from Health Camp Participants; S3: Questionnaire.

## Abbreviations

The following abbreviations are used in this manuscript:

CCEs	Community Cancer Educators
NCCP	National Cancer Control Program
WWC	Well Woman Clinic
CCMB	CancerCare Manitoba
LMICs	Low- and Middle-Income Countries
MOH	Medical Officers of Health
CEDC Jaffna	Centre for Early Detection of Cancer Jaffna
CANE	Cancer Aid North East
