# Supplementary material for: A Report from a Community-Centric Cancer Control Approach in the Post-Conflict Northern Province of Sri Lanka
Source: Int J Environ Res Public Health. 2025 Sep 27;22(10):1492. doi: 10.3390/ijerph22101492 (PMC12562639; doi:10.3390/ijerph22101492)
Supplement: Supplementary file 1 [file ijerph-22-01492-s001.zip › ijerph-3720586-supplementary.pdf]

International Journal of
Environmental Research and Public Health

MDPI

Supplementary to Article

Report from a Community-Centric Cancer Control Approach in Post-Conflict Northern Province of Sri Lanka.

Abiola N Dosumu [1], Antony. J. Thanenthiran [2], Ganeshamoorthy Sritharan [2], Thanuja Mahendran [2], Rajendra Surenthirakumaran [3], Kandasamy Sithamparanathan [4], Stephanie Asence [1], Kathleen M. Decker [1,5] and Sri Navaratnam [1,6]

S1: Health Camp Participants: District- Level Summary

		Jaffna (181)	Kilinochchi (168)	Mannar (131)	Mullaitivu (130)	Vavuniya (237)
**Age**	Below 30	23 (12.7%)	25 (14.9%)	3 (2.3%)	29 (22.3%)	9 (3.8%)
	30-65	120 (66.3%)	116 (69.0%)	109 (83.2%)	87 (66.9%)	193 (81.4%)
	Above 65	38 (21.0%)	27 (16.1%)	19 (14.5%)	14 (10.8%)	35 (14.8%)
**Sex**	Male	54 (29.8%)	56 (33.3%)	38 (29.0%)	44 (33.8%)	61 (25.7%)
	Female	127 (70.2%)	112 (66.7%)	93 (71.0%)	86 (66.2%)	176 (74.3%)
**Occupation**	Housewife	29 (16.0%)	10 (6.0%)	36 (27.5%)	25 (19.2%)	99 (41.8%)
	Craft and trade workers	28 (15.5%)	20 (11.9%)	14 (10.7%)	25 (19.2%)	45 (19.0%)
	Agricultural workers	18 (9.9%)	9 (5.4%)	9 (6.9%)	24 (18.5%)	7 (3.0%)
	Professionals	8 (4.4%)	9 (5.4%)	11 (8.4%)	9 (6.9%)	8 (3.4%)
	Students	6 (3.3%)	6 (3.6%)	3 (2.3%)	1 (0.8%)	6 (2.5%)
	Retirees	0 (0.0%)	1 (0.6%)	2 (1.5%)	0 (0.0%)	1 (0.4%)
	Unknown/ not reported	92 (50.8%)	113 (67.3%)	56 (42.7%)	46 (35.4%)	71 (30.0%)

Table S1: Characteristics of participants at community health camps in Sri Lanka's Northern Province, by district.

S2: Testimonials from Health Camp Participants.
Participants included local community members who attended the camp, as well as healthcare staff involved in delivering services and education. These testimonials highlight personal experiences, perceptions, and the impact of the health camp on the community.

Participant A: "I am so enthusiastic because of the friendly environment that has been created."

Participant B: "It was my fear of stigma that stopped me from participating in cancer screening. This is the first time a team of doctors, educators, and activists reached out to us."

Participant C: "In this environment, it is easier to express and share my fears. We feel much more confident about going to a hospital for treatment."

Participant D: "This day is historic for our community. The real cause of sadness and death, I believe, is ignorance, but today a seed has been planted to make our lives easier. It is also the first time a hospital has come to our doorstep; usually, we have to go to the hospital ourselves. This gives us a new level of confidence."

Cancer Nurse Participant: "So far, we have only treated patients who came to the teaching hospital in Jaffna. This is a unique experience where we actually travel to where the patients live and bring the hospital to the community."

S3: Questionnaire

Cancer Early Detection Center Jaffna

E-mail: cedc.nccp.jaf@gmail.com

<table>
<tr><th colspan="6">Community Screening Questionnaire</th></tr>
<tr><th colspan="6">Biodata</th></tr>
<tr><td colspan="4">Registration No:</td><td colspan="2">Date:</td></tr>
<tr><td colspan="4">Age:</td><td colspan="2">Sex: Male/ Female</td></tr>
<tr><td colspan="4">Social Status: Married / Unmarried</td><td colspan="2">Children: Yes/No</td></tr>
<tr><td colspan="6">Address: (District and Village)</td></tr>
<tr><td colspan="6">Telephone No:</td></tr>
<tr><td colspan="6">Occupation:</td></tr>
<tr><td colspan="6"></td></tr>
<tr><th colspan="6">Medical History</th></tr>
<tr><td colspan="4">PMHx</td><td colspan="2">PSHx</td></tr>
<tr><td colspan="6">Previous Pap Smear: (Date and Result)</td></tr>
<tr><td colspan="6">Previous HPV DNA: (Date and Result)</td></tr>
<tr><td colspan="6">Previous Mammogram/USS Scan: (Date and Result)</td></tr>
<tr><td colspan="6"></td></tr>
<tr><th colspan="6">Risk Factors</th></tr>
<tr><td colspan="4">Age of Menarche</td><td colspan="2">LRMP:</td></tr>
<tr><td colspan="6">Birth Control: (OCP/ HRT/ Others)</td></tr>
<tr><td colspan="4">Age of Menopause</td><td colspan="2">Post- Menopausal Bleeding:</td></tr>
<tr><td>Betel Quid Chewing</td><td colspan="3">Yes:</td><td colspan="2">No:</td></tr>
<tr><td>Tobacco Consumption</td><td colspan="3">Yes:</td><td colspan="2">No:</td></tr>
<tr><td>Alcohol Consumption</td><td colspan="3">Yes:</td><td colspan="2">No:</td></tr>
<tr><td>Occupation Radiation</td><td colspan="3">Yes:</td><td colspan="2">No:</td></tr>
<tr><td>Exposure to Chemicals</td><td colspan="3">Yes:</td><td colspan="2">No:</td></tr>
<tr><td rowspan="4">Family History of Cancer</td><td colspan="5">No:</td></tr>
<tr><td>Yes</td><td colspan="4">Degree of Relationship</td></tr>
<tr><td rowspan="2">Type of Cancer</td><td>1st Degree</td><td>2nd Degree</td><td>3rd Degree</td><td>Not Known</td></tr>
<tr><td></td><td></td><td></td><td></td></tr>
<tr><th colspan="6">Examination</th></tr>
<tr><td colspan="6">Oral (Normal/Abnormalities):</td></tr>
<tr><td colspan="6">Lymph Nodes:</td></tr>
<tr><td colspan="6">Breast: (Normal/Abnormalities):</td></tr>
<tr><td colspan="6">Referred (Yes/ No)</td></tr>
</table>

PMHx: Past Medical History

PSHx: Past Surgery

USS: Ultrasound

OCP: Oral Contraceptive Pill

HRT: Hormonal Replacement Therapy

LRMP: Last Regular Menstrual Period

C.Referral form

COMMUNITY CANCER AWARENESS & SCREENING

Referral Note

PATIENT INFORMATION

Date :

Village :

Date of birth : Age: Sex

Male/Female

Telephone :

MEDICAL HISTORY

Diagnosis

ADDITIONAL INFORMATION

Referred to:

Referred by:

Onco Surgical Unit
Jaffna teaching Hospital
021-2222640 070-7772640

JMFOA CANADA

CANE
